# Instabilities of Thin Films on a Compliant Substrate: Direct Numerical Simulations from Surface Wrinkling to Global Buckling

**DOI:** 10.1038/s41598-020-62600-z

**Published:** 2020-03-31

**Authors:** Siavash Nikravesh, Donghyeon Ryu, Yu-Lin Shen

**Affiliations:** 1 Department of Mechanical Engineering, University of New Mexico, Albuquerque, NM 87131 USA; 2 Department of Mechanical Engineering, New Mexico Institute of Mining and Technology, Socorro, NM 87801 USA

**Keywords:** Mechanical engineering, Computational methods

## Abstract

For structures consisting of a thin film bonded to a compliant substrate, wrinkling of the thin film is commonly observed as a result of mechanical instability. Although this surface undulation may be an undesirable feature, the development of new functional devices has begun to take advantage of wrinkled surfaces. The wrinkled structure also serves to improve mechanical resilience of flexible devices by suppressing crack formation upon stretching and bending. If the substrate has a reduced thickness, buckling of the entire structure may also occur. It is important to develop numerical design tools for predicting both wrinkle and buckle formations. In this paper we report a comprehensive finite element-based study utilizing embedded imperfections to directly simulate instabilities. The technique overcomes current computational challenges. The temporal evolution of the wrinkling features including wavelength and amplitude, as well as the critical strains to trigger the surface undulation and overall structural buckling, can all be predicted in a straightforward manner. The effects of model dimensions, substrate thickness, boundary condition, and composite film layers are systematically analyzed. In addition to the separate wrinkling and buckling instabilities developed under their respective geometric conditions, we illustrate that concurrent wrinkling and buckling can actually occur and be directly simulated. The correlation between specimen geometry and instability modes, as well as how the deformation increment size can influence the simulation result, are also discussed.

## Introduction

**Figure 1:**
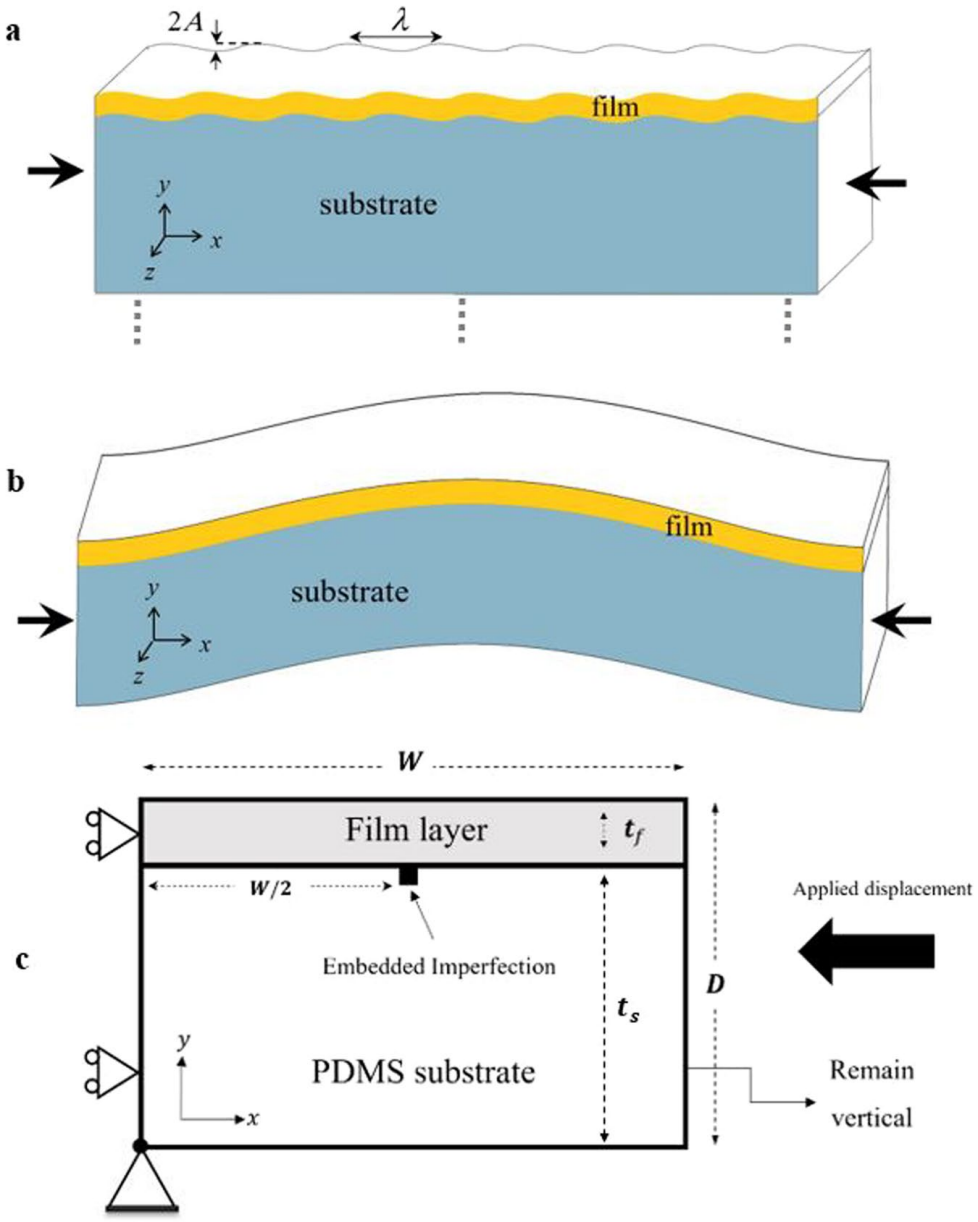
Schematics of (**a**) local surface wrinkles and (**b**) global buckling of the film-substrate structure during compressive loading along the *x*-axis. (**c**) Geometric parameters and boundary conditions used for the simulations.

Formation of wrinkles can occur on a thin film bonded to a thick compliant substrate. When in-plane compression in an initially flat structure reaches a certain extent due to mechanical loading, thermal expansion mismatch, or chemical reactions, wrinkling partially diminishes the strain energy in the film layer and relieves compressive strain. A schematic of a wrinkled surface is shown in Fig. 1a. The instability-driven surface undulation, while frequently undesirable, may also be exploited to improve device functionalities. For instance, surface wrinkles can improve the luminous efficiency associated with organic light emitting tools^1,2^ and light harvesting efficiency of organic photovoltaic (PV) layers^2–4^. For stiff inorganic conducting and photovoltaic layers (for instance, perovskite, silicon, and indium-tin oxide (ITO)), several methods for fabrication onto a wavy substrate structure, for the purpose of improving trapping^5–8^ and scattering^9^ of sunlight, have also been reported. A wrinkled structure can lead to improved deformability in stretchable electronics^10,11^, thus enabling their deployment on curved surfaces and in fabrics. Their mechanical resilience with respect to stretching, bending, and cyclic loading have been demonstrated while preserving tenable optoelectronic performances^12–14^. The development of various self-organizing mechanisms is also a direct application of wrinkling instability^15–20^.

Surface wrinkles can be produced across a wide area with relative ease. A common approach is to fabricate the film layer on a pre-tensioned elastomeric material. The film is effectively under compressive strain upon releasing the pre-stretch; surface instability will be induced if the compressive strain experienced by the film exceeds a critical value, so as to decrease the strain energy of the system. In cases where the substrate is thinner (far from being considered as semi-infinite), buckling of the entire structure may also occur which will influence the wrinkling behavior. Figure 1b schematically shows a buckled film-substrate structure. It is important to be able to predict and control the instabilities, which requires robust numerical tools with the capability of handling various material/geometric characteristics related to the thin film-substrate systems. While theoretical solutions exist for both the surface wrinkling and overall buckling instabilities (key equations to be presented in the following section), they were predicated upon idealized conditions, and the interplay between wrinkling and buckling is not altogether clear.

Note that in the literature the term buckling was sometimes used to represent the wrinkling behavior. In the present work the term buckling is strictly used to describe the formation of a global wave form of the entire structure as shown in Fig. 1b. The term wrinkling refers to the local surface wave form displayed by the thin films as shown in Fig. 1a.

Numerically simulating deformation instabilities using the finite element technique is inherently challenging. A common practice to simulate the surface wrinkling phenomenon is through a multi-step process including pre-instability and post-instability analyses^21–24^. The pre-instability step consists of a linear modal analysis, and the post-instability analysis was conducted by employing imperfection techniques in combination with various nonlinear geometric/material models. As an example of the material imperfection approach, a non-uniform plastic model was utilized for an elastoplastic film^25^. The geometric imperfection techniques, employed more often for triggering surface wrinkling modes, may incorporate the geometry, boundary condition, and mesh perturbation techniques^21,22,26,27^. All these approaches are complex and computationally intensive, and they apparently build the waves into the model and constrain the subsequent formation of instabilities.

The interaction between the global and local deformation instabilities have been historically studied for thin-walled structures, slender columns, and sandwich panels^28–32^. It has been analytically viewed as a “competition” between the formation of either the global or local modes of deformation; the possibility of concurrent wrinkling-buckling instability, starting from a fully flat geometry, was generally ignored. Moreover, many of the available numerical/analytical studies related to global/local instabilities in thin-walled structures and sandwich panels were based on the direct incorporation of geometric imperfections within the model^28,31,33–35^. The imperfection dependency of the solution was well recognized. Frequently two types of geometric imperfections, namely global imperfection (for activating buckling modes) and local imperfection (for activating wrinkling modes), were utilized within the model^35^ which is an apparent example of building a wave form into the numerical framework, and fully control/constrain the subsequent deformation instability response.

The current paper presents a straightforward and robust finite element modeling strategy to numerically simulate the formation and development of deformation instabilities. In this approach, termed “embedded imperfections,” perturbed elastic properties are assigned to one or more regular elements at the interface of the film-substrate structure, for the purpose of triggering the formation of wrinkles without the need of any multi-step process. This work is originated from our recent development^36,37^, but is now expanded into a comprehensive study of the effects of model dimensions and boundary condition, using only one embedded imperfection, to fully assess the modeling capability. Most importantly, we illustrate that the formation of surface wrinkles and overall structural buckles can both be predicted from a single numerical implementation, and that concurrent wrinkling and buckling can actually occur and be directly simulated. The main objectives of this paper are thus to comprehensively study (1) the periodic unit-cell approach based on placement of only one embedded imperfection and (2) the “concurrent buckling-wrinkling” deformation instability mode in film-substrate structures, to unveil the full potential of the modeling technique.

In the presentation below a theoretical overview is first provided, which is followed by the numerical model description and verification. Systematic analyses of the effects of model width, substrate thickness, boundary condition, and composite film layers are then presented. Discussions on the correlation between the geometric conditions and instability modes, as well as how the deformation increment size can influence the simulation result, are also included.

## Theory overview

In this section, some of the well-established analytical solutions associated with surface wrinkling of semi-infinite elastic film-substrate structures, and global buckling of their finite-size counterparts, are presented. These relations are used for the verification of the numerical models and serve as the basis from which deviation can be quantified through the more realistic numerical simulations in subsequent sections.

Consider a thin film-compliant substrate structure under the applied compression shown in Fig. 1a. The planar elastic solutions for the critical wavelength (*λ*_*cr*_), the amplitude (*A*), and the critical wrinkling strain (*e*_*cr*_) have been derived^38–42^ as follows

1 $${\lambda }_{cr}=2\pi {t}_{f}{\left[\frac{(1-{\nu }_{s}^{2}){E}_{f}}{3(1-{\nu }_{f}^{2}){E}_{s}}\right]}^{\frac{1}{3}},$$

2 $${e}_{cr}={({e}_{xx})}_{cr}=\frac{1}{4}{\left[\frac{3(1-{\nu }_{f}^{2}){E}_{s}}{(1-{\nu }_{f}^{2}){E}_{f}}\right]}^{\frac{2}{3}},$$

3 $$A=\left(\frac{{\lambda }_{cr}}{\pi }\right){(e-{e}_{cr})}^{\frac{1}{2}}={t}_{f}{\left(\frac{e}{{e}_{cr}}-1\right)}^{\frac{1}{2}};$$

where *E*_*f*_  and *E*_*s*_ are, respectively, Young’s moduli of the thin film and substrate; *v*_*f*_ and *v*_*s*_ are, respectively, Poisson’s ratios of the film and substrate, *t*_*f*_ is the thickness of the film (single-layer), and *e* is the applied compressive strain (*e* = *e*_*xx*_). Note that *λ*_*cr*_ in Eq. (1) is the starting wavelength and thus independent of the applied strain *e*. The strain-dependent wavelength of uniformly developed wrinkles, *λ*, can be written as^43,44^

4 $$\lambda ={\lambda }_{cr}[1-(e-{e}_{cr})].$$

Equations (1–3) were derived based on a thick substrate (semi-infinite) under the assumptions of plane strain compression, isotropic linear elasticity, negligible tangential stress at the film-substrate interface, uniform wrinkling waves throughout the entire film span, and perfectly bonded layers.

For the same type of structure, a thin film above a compliant substrate, a different form of instability, namely global buckling as depicted in Fig. 1b, may occur if the substrate thickness is significantly reduced. Using the elastic composite beam theory under the plane strain condition, the effective axial rigidity, $\overline{EA}$, and effective bending rigidity, $\overline{EI}$, of the equivalent composite beam are written^45,46^ as

5 $$\overline{EA}={\bar{E}}_{s}{t}_{s}+{\bar{E}}_{f}{t}_{f},$$

and

6 $$\overline{EI}=\frac{{({\bar{E}}_{f}{t}_{f}^{2}-{\bar{E}}_{s}{t}_{s}^{2})}^{2}+4({\bar{E}}_{f}{\bar{E}}_{s})({t}_{f}{t}_{s}){D}^{2}}{12\overline{EA}};$$

where *D* is defined as the total film/substrate thickness (*D* = *t*_*s*_ + *t*_*f*_), and ${\bar{E}}_{f}$ and ${\bar{E}}_{s}$ are the plane-strain moduli, i.e., ${\bar{E}}_{f}={E}_{f}/(1-{\nu }_{f}^{2})$ and ${\bar{E}}_{s}={E}_{s}/(1-{\nu }_{s}^{2})$. Accordingly, for an equivalent composite beam of width (length) *w* with clamped supports, the critical buckling strain is^45,47^

7 $${({e}_{cr})}_{b}=\left[\frac{{({F}_{cr})}_{b}}{\overline{EA}}\right]\left[\frac{1}{1+1.2{({F}_{cr})}_{b}/(\bar{G}D)}\right],$$

where (*F*_*cr*_)_*b*_ is the critical buckling force defined as ${({F}_{cr})}_{b}=4{\pi }^{2}\overline{EI}/{w}^{2}$, and $\bar{G}$ is the effective shearing modulus following the inverse rule of mixture as $1/\bar{G}={t}_{f}/(D{G}_{f})+{t}_{s}/(D{G}_{s})$. Note that the shear effects have been neglected in the derivation of (*F*_*cr*_)_*b*_ in Eq. (7).

It has been postulated that the smaller of the critical strains, either the surface wrinkling critical strain in Eq. (2), or the global buckling critical strain in Eq. (7), controls the deformation mode^48,49^. By setting the condition (*e*_*cr*_)_*b*_ = *e*_*cr*_, the critical width *w*_*cr*_, which separates the global buckling and local surface wrinkles, is obtained as

8 $${w}_{cr}=4\pi \sqrt{\overline{EI}\left[\frac{{({\bar{E}}_{f}/(3{\bar{E}}_{s}))}^{2/3}}{\overline{EA}}-\frac{0.3}{({G}_{s}D)}\right]}.$$

It was perceived that global buckling will occur if *w* > *w*_*cr*_, and local wrinkling will occur if *w* < *w*_*cr*_. Although the configuration of local wrinkles developed on a globally bent structure has recently been proposed^48–50^, theoretical considerations in the literature have generally ignored the possibility that wrinkling and buckling can codevelop, starting from a fully flat geometry. It will be shown in the following sections that a transitional state (with both wrinkles and global buckle) can be captured, by employing the current direct numerical simulation approach.

## Numerical model description

Figure 1c shows the modeling domain along with the boundary and loading conditions used in the simulations. The model was constructed for systematically studying the effects of its dimensions on the development of instabilities. Both the substrate and the thin film were taken to be isotropic linear-elastic solids, consisting of eight-noded continuum quadrilateral elements. The generalized plane strain condition^51^, accounting for the uniform out-of-plane (*z*-direction) deformation, is considered. In some specific cases the classical 2D plane strain condition is also used, for the purpose of illustrating its difference from the more realistic generalized plane strain condition. The model domain may be viewed as a repetitive segment of a larger periodic system along *x*-direction. As shown in Fig. 1c, a symmetry boundary is considered on the left edge with the *x*-direction displacement prohibited. The lower-left corner point is not allowed to move in *x* and *y*. A uniform compressive displacement (in negative *x*) is imposed on the right edge so it remains vertical during deformation^52^. In addition, rotations about the *x*- and *y*-axes are not allowed under for the current generalized plane strain condition. All simulations were conducted using the finite element software ABAQUS (Dassault Systems Simulia Corp., Johnston, RI, USA, Version 2017), under the static condition and geometric nonlinearity (large deformation analysis).

The compliant substrate considered in this study is PDMS (polydimethylsiloxane), with elastic modulus *E*_*s*_ = 2.97 MPa^53^ and Poisson’s ratio of *v*_*s*_ = 0.495 (taken to be slightly lower than 0.5 to eliminate potential convergence issues). Two thin film materials are considered: P3HT:PCBM (a p-n semiconducting polymer blend of regioregular poly-3-hexylthiophene conjugated polymer and phenyl-C61-butyric acid methyl ester fullerene derivative) with elastic modulus *E*_*f*_ = 7300 MPa, and PEDOT:PSS (poly-3,4-ethylenedioxythiophene and polystyrene sulfonate acid) with *E*_*f*_ = 2000 MPa^54^. Their Poisson’s ratios are both taken as *v*_*f*_ = 0.35. In some of the figures presented below, P3HT:PCBM is referred to as P3HT and PEDOT:PSS is referred to as PEDOT for the purpose of brevity. In addition to the single-layer film, limited cases of composite (bilayer) films on the PDMS substrate is also considered, with equal thicknesses of P3HT:PCBM (top layer) and PEDOT:PSS (in contact with the substrate). Note that this layered structure is identical to those reported for organic flexible PV cells^12^. It also serves as a portion of the mechano-optoelectronic strain sensing device under development^14^. Detailed information about the composite film model will be given in a later section.

Throughout the study the film’s thickness is kept constant at *t*_*f*_ = 0.1 *μm*, while the width (*w*) and depth (*D*) of the structure are systematically varied. The width and depth ranges studied are summarized in Table 1, with the simulated cases marked with an asterisk. Note that both *w* and *D* are expressed in terms of critical wrinkling wavelength (Eq. (1), different values for different film materials), as will be discussed further in the following sections. Unless otherwise stated, this scaling representation is used throughout the paper.

**Table 1:** The variation of depth and width considered for the numerical simulations.

		*D*										
		1*λ*	2*λ*	4*λ*	5*λ*	10*λ*	20*λ*	50*λ*	80*λ*	100*λ*	120*λ*	1000 *μm*
*w*	2*λ*											*
	5*λ*											*
	10*λ*	*	*	*	*	*	*	*	*	*	*	*
	20*λ*	*	*	*	*	*	*	*	*	*	*	*
	40*λ*	*	*	*	*	*	*	*	*	*	*	*
	80*λ*											*
	110*λ*											*

The embedded imperfection approach^36,37^ is used for triggering instabilities. The embedded imperfections are selected regular finite elements immediately below the perfectly bonded film/substrate interface (in the substrate layer). These elements carry material properties the same as the film material. The applicability of this approach in generating wrinkling instability was demonstrated in our previous studies, first for single-layer thin film/thick substrate systems discretized by four-noded linear elements^36^ and then extended to composite-film/thick substrate systems with higher order eight-noded elements^37^. In the present work, a thorough investigation of model geometry from thick to thin substrates and from large to small widths, are undertaken, to demonstrate the full capability of the numerical approach. Furthermore, we aim to embed only one imperfection at the center of the interface, as shown in Fig. 1c, and capture the full modes of instabilities from local wrinkling to global buckling in a seamless manner.

## Model verification

**Figure 2:**
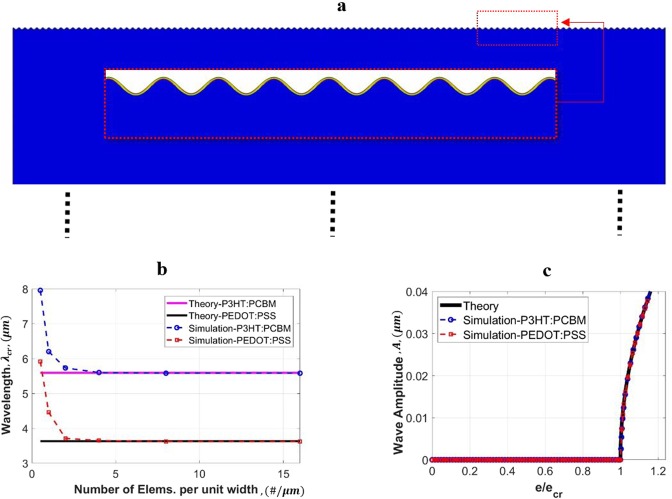
(**a**) A simulated wrinkling configuration used for the verification study, with *D* = 1000 *μm* and *w* = 110*λ* (this image pertains to the result of the finest mesh). (**b**) The wrinkling wavelength (*λ*_*cr*_) as a function of mesh density. (**c**) The wrinkling amplitude (*A*) as a function of normalized applied strain (*e*/*e*_*cr*_). Theoretical solutions are also included in the presentation.

Extensive preliminary simulations were conducted to establish mesh independence, focusing on the formation of surface wrinkles, which is a more severe form of deformation (than global buckling) and requires finer meshes. A typical wrinkling configuration is shown in Fig. 2a using the 0.1 *μ*m-thick P3HT:PCBM film on the PDMS substrate, with *D* = 1000 *μm* and *w* = 110*λ* (with *λ* equal to 5.5919 *μm*). The model size is large enough to represent a semi-infinite substrate, but only the top portion of the substrate in shown. Note that in this figure and all the others showing wrinkles and/or buckles, a deformation amplification factor is utilized for the post-processed images to make the wave form more evident. Figure 2b shows the variation of critical wrinkle wavelength with the mesh density, represented by the number of elements per unit domain width. In addition to the P3HT:PCBM film, the result of the single-layer PEDOT:PSS film is also included. The figure also includes horizontal lines which are analytical solutions based on Eq. (1). The numerical results converge to theoretical solutions in both cases providing that the mesh is sufficiently fine.

Figure 2c shows the evolution of the amplitude as a function of the applied compressive strain. Here the finest mesh size presented in Fig. 2b was used, and the amplitude is normalized by the critical strain value. Figure 2c also includes the theoretical solution, Eq. (3), which does not depend on the film material. The wrinkle amplitude stays as zero until the critical point; at the onset of instability the amplitude starts to increase. The numerical results match the analytical solution very well. Note that there is no cumbersome multi-step process involved in the simulations. The formation of wrinkles and their subsequent evolution can be obtained in a straightforward and seamless manner.

All numerical results in the remainder of this paper are obtained with the finest mesh size reported in Fig. 2b. Also, across the film thickness there are at least four uniform layers of elements. Depending on the depth of the problem domain (substrate thickness), adapted mesh distribution with finer elements near the film interface is employed in the substrate. In all cases only one embedded imperfection is placed at the center of the film-substrate interface.

## Surface wrinkling: Effects of domain width

The model geometry used for the results in the previous section has the greatest width and depth studied in this paper, i.e., the bottom case in the right-most column in Table 1. In this section, we explore surface wrinkle formation by moving up the column with decreasing widths while keeping *D* = 1000 *μm*. The choice of the greatest width in this study, 110λ, is worthy of elaboration. It is based on our prior finding^36,37^ that, if more than one imperfections are to be included in the model, their maximum spacing, when normalized by the wavelength, should not be greater than 55 in order to generate uniform sinusoidal wrinkles conforming to the theoretical solution. As a consequence, to include only one central imperfection in the present study for making the model a “representative volume element,” a total width of 110λ (55λ on each side of the imperfection) is the maximum allowed. The numerical result in the previous section illustrated that this choice works very well as the starting model width.

**Figure 3:**
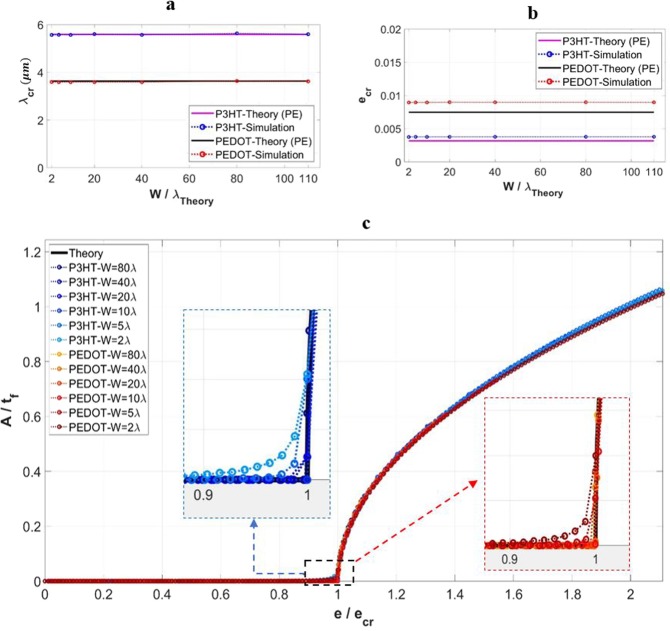
Variation of the simulated (**a**) critical wrinkling wavelength (*λ*_*cr*_), and (**b**) critical wrinkling strain value (*e*_*cr*_) with the domain width, with the width normalized by the theoretical critical wrinkling wavelength value (*w*/*λ*). (**c**) The normalized wrinkling amplitude (*A*/*t*_*f*_) as a function of normalized applied strain (*e*/*e*_*cr*_), for different widths and film properties. Theoretical solutions are also included in the presentation.

Figure 3a,b show the simulated critical wrinkle wavelength and the critical strain to trigger wrinkles, respectively, as a function of the domain width normalized by the theoretical wavelength, for the cases of single-layer films of P3HT:PCBM and PEDOT:PSS. For comparison the theoretical values are also shown. As can be seen, the numerically calculated wavelength and critical strain are both essentially independent of the domain width. The wrinkling wavelength matches the theoretical value. However, the simulated critical strain values are higher than the analytical solutions for both film materials. Recall that the theories pertain to the plane strain (PE) assumption but the more realistic condition, namely generalized plane strain (GPE), is incorporated in the simulations. While the GPE prediction of wavelength is the same as PE, GPE allows the Poisson expansion in the *z*-direction when the model is compressed in the *x*-direction, which prolongs the pre-instability (flat) deformation. As a consequence the critical strain for instability becomes higher. Note that if PE is used in the numerical modeling, the predicted critical strain also agrees with the analytical solutions^37^.

Figure 3c presents the surface wrinkling amplitude as a function of the applied strain, for the two film materials with various domain widths. In the figure the normalized amplitude (by the film thickness) and the normalized applied strain (by the numerically obtained critical value) are used. It is evident that the numerical response is generally insensitive to the domain width, and thus all curves are close to the theoretical one. However, there is a subtle effect when the applied strain approaches the critical point as illustrated in the insets in Fig. 3c. For models with a very small width, the triggering of instability appears to be more gradual rather than abrupt. This is due to the close distance between the imperfection and the domain edge. Since wrinkling always starts from the small disturbance around the imperfection shortly before the critical strain is reached, the wavy feature thus easily spread through the entire width of a small model, leading to an apparent early start of the instability. Nevertheless, the evolving wrinkle amplitude soon merges into the same response of all the other cases.

The results presented above suggest that one does not need a very wide simulation domain to obtain the wrinkling behavior. A small model can adequately yield reliable wrinkling parameters using the current approach.

## Effects of domain depth

Attention is now turned to the effects of model depth *D*, with reference to the problem domain shown in Fig. 1c. Numerous models were generated for a comprehensive study of depth dependency of instabilities in the film/substrate system. Here three different widths are considered: *w* = 10*λ*, 20*λ*, and 40*λ*, and the depth is varied from the thickest of *D* = 1000 *μ*m to as thin as *D* = 1*λ* as seen in Table 1, the middle three rows. Since the film thickness is fixed at 0.1 *μ*m in this study, the variation of *D* is entirely determined by substrate thickness.

**Figure 4:**
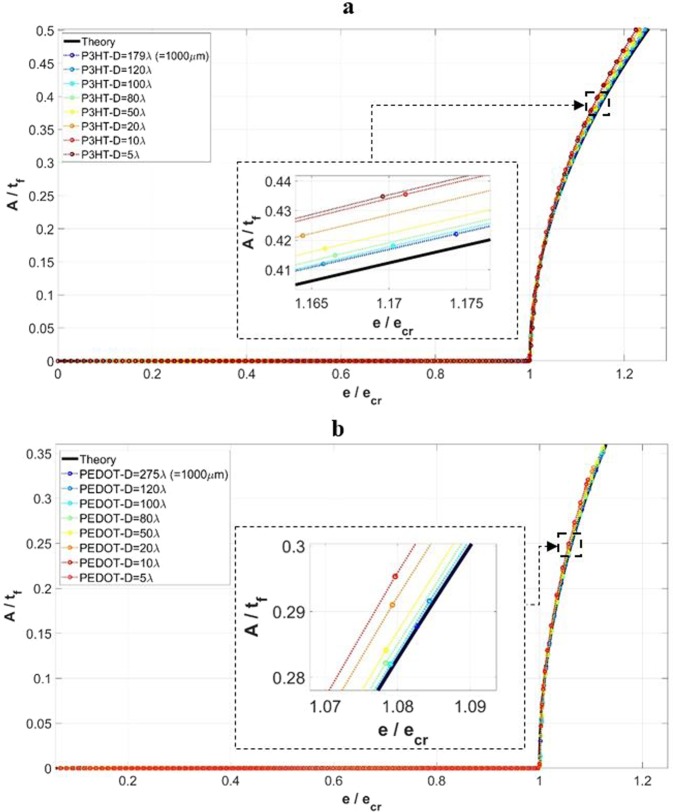
The normalized wrinkling amplitude (*A*/*t*_*f*_) as a function of normalized applied strain (*e*/*e*_*cr*_), for the width of w = 40λ and domain depth sizes of *D* ≥ 5*λ*. The film materials considered are (**a**) P3HT:PCBM, and (**b**) PEDOT:PSS. Theoretical solutions are also included in the presentation.

Consider first the case of *w* = 40*λ*. It was observed from all the simulation results that for the depth sizes of *D* ≥ 5*λ*, uniform surface wrinkles will form. In Fig. 4, the numerically obtained surface wrinkling amplitude is plotted against the applied strain, for the depth range of *D* ≥ 5*λ*. In the presentation the amplitude is normalized by film thickness, and the applied strain is normalized by the critical value. The two cases of single-layer P3HT:PCBM film (Fig. 4a) and PEDOT:PSS film (Fig. 4b) are considered. The analytical solution, Eq. (3), is also incorporated in the figure. It can be seen that, as the domain depth decreases, the simulated amplitude deviates from the theoretical response and becomes slightly higher. The difference, however, is very small within the strain range of $\frac{e}{{e}_{cr}}\le 1.20$.

**Figure 5:**
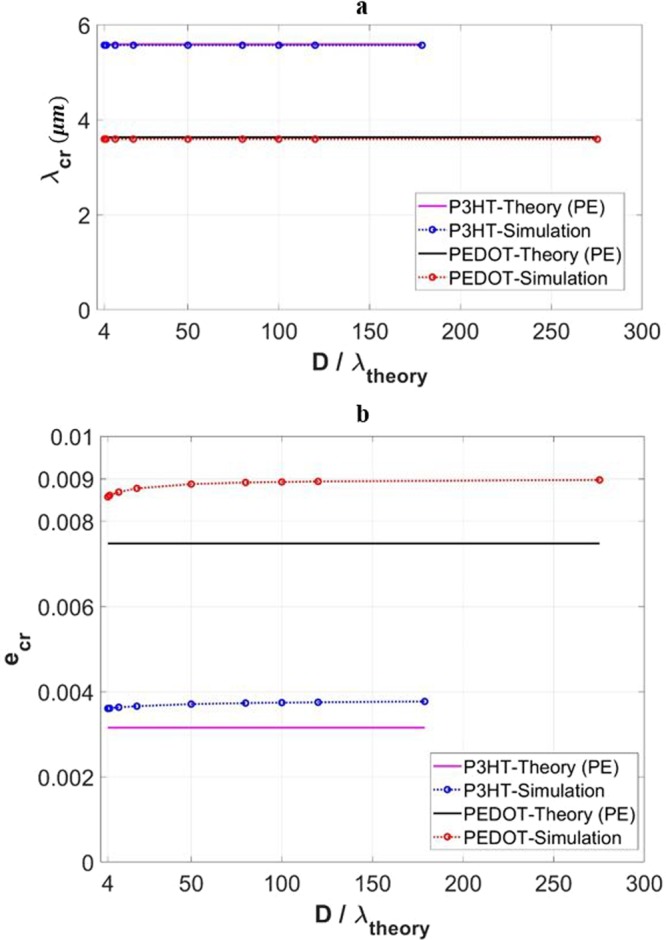
Variations of (**a**) critical wrinkling wavelength (*λ*_*cr*_), and (**b**) critical wrinkling strain (*e*_*cr*_) with the normalized domain depth (*D*/*λ*), for the cases of *D* ≥ 4*λ* and *w* = 40*λ*. Results for the P3HT:PCBM film and PEDOT:PSS film are included. Theoretical solutions are also included in the presentation.

Figure 5a,b show the simulated critical wrinkling wavelength and critical strain, respectively, as a function of the domain depth normalized by the analytical wavelength. The range of domain depth considered is above 4*λ*. The theoretical values are plotted as the horizontal lines. The critical wavelength, Fig. 5a, remains constant and matches the theoretical values well for both the two cases of film materials. As for the critical strain, Fig. 5b, the simulation results are higher than the plane-strain theoretical values for reasons discussed in the previous section. The predicted critical strain shows a small reduction as the depth becomes small. Note that this slight reduction of critical wrinkling strain can also be seen by carefully examining the several numerical datapoints reported in other earlier studies^10,45^.

**Figure 6:**
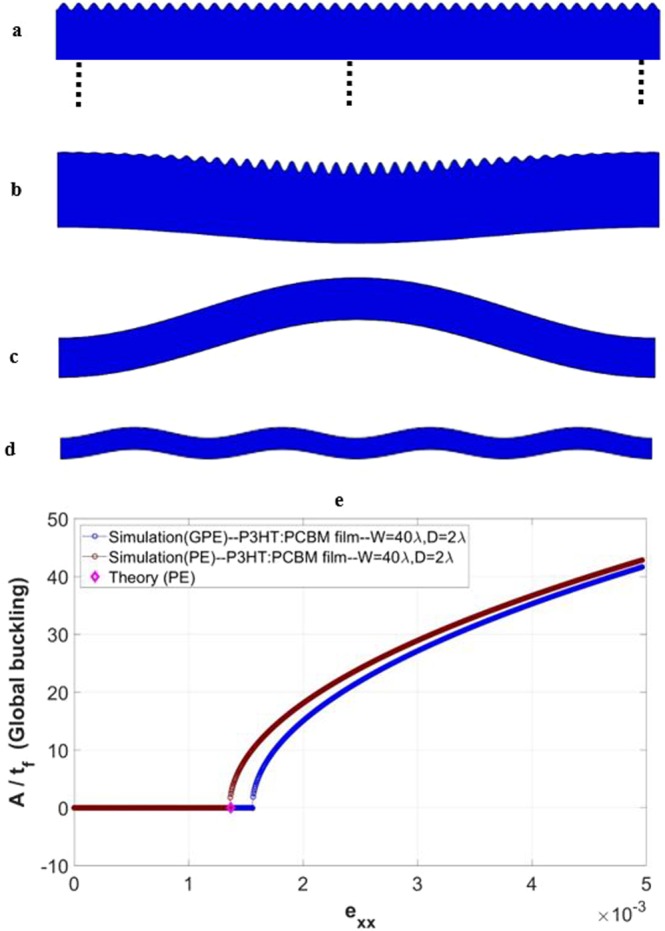
(**a**–**d**) Schematics showing the numerically obtained instability modes for *w* = 40*λ*, and (**a**) *D* ≥ 5*λ*, (**b**) *D* = 4*λ*, (**c**) *D* = 2*λ*, (**d**) *D* = *λ*, in the case of P3HT:PCBM film. (**e**) Variation of the normalized global buckling amplitude with applied strain, associated with (**c**) and including the GPE and PE simulation response. The theoretical global buckling critical strain value (plane strain) is also included for comparison.

As mentioned above, we observed that local wrinkling occurs when the domain depth *D* ≥ 5*λ*. Although at 5*λ* the depth is drastically reduced from the greater values in the right-hand part of Table 1, the substrate is still sufficiently “thick” so surface wrinkling is the only form of instability that can occur. (Take the P3HT:PCBM thin film for instance, at *D* = 5*λ* the ratio of *t*_*s*_/*t*_*f*_ is still approximately 280. At *w* = 40*λ*, the aspect ratio of the entire structure, *w*/*D*, is 8.) A typical form of surface wrinkles for the cases of *w* = 40*λ* and *D* ≥ 5*λ* is shown in Fig. 6a. For clarity the image only includes the P3HT:PCBM film and the very top portion of the PDMS substrate. As the depth is reduced to 4*λ*, a different form of instability appears: local wrinkling *in conjunction with* global buckling as shown in Fig. 6b. The slender overall geometry (*w*/*D* = 10) now triggers buckling. Figure 6b clearly illustrates that local wrinkling and global buckling are not mutually exclusive.

With further reductions of depth to *D* ≤ 2*λ*, surface wrinkles cease to exist, and global buckling becomes the only form of instability observed, as shown in Fig. 6c for *D* = 2*λ* and in Fig. 6d for *D* = *λ*. The occurrence of global buckling shows consistency with the theories. Consider for example the case of *w* = 40*λ* and *D* = 2*λ* with the P3HT:PCBM film. The theoretical critical width, calculated from Eq. (8), is ${w}_{cr}=132.15\,\mu m$. With $w=40\lambda =223.67\,\mu m > {w}_{cr}$, the global buckling condition is satisfied. Figure 6e shows the variation of the normalized global buckling amplitude as a function of the applied strain, in the case of P3HT:PCBM film with *w* = 40*λ* and *D* = 2*λ*. The significance of Fig. 6e is that, in addition to local wrinkling, global buckling can also be directly simulated with the current embedded imperfection technique. The pre-buckle stage (regular compression), onset of global buckling, and post-buckle behavior can all be implemented in a single analysis step in a straightforward manner. In Fig. 6e numerical results based on both GPE and PE conditions are included, as well as the theoretical critical buckling strain (a single value), Eq. (7). As can be seen, the simulated onset of buckling (under PE condition) coincide with the theoretical critical buckling strain. The GPE condition results in a delayed critical state, similar to the case of local wrinkling discussed previously.

The coexistence of local surface wrinkling and global buckling, found when the depth is within the range 2*λ* < *D* < 5*λ* for *w* = 40*λ*, as revealed in Fig. 6b for the case of P3HT:PCBM model with *D* = 4*λ*, deserves further discussion. The formation of surface wrinkles starts first, with the wavelength and critical strain shown in Fig. 5a,b, respectively. These numerical values follow the same trend as those of thicker substrates. With further straining in this case, however, an overall buckled shape starts to emerge while the uniform wrinkles remain in place. The existence of such transitional behavior, between pure local wrinkling and pure global buckling, was not taken into account in typical analytical considerations^45,55^ and has not been captured by other numerical means. It is worthy of examining this behavior in more detail. In the case of *w* = 40*λ* and *D* = 4*λ* with the P3HT:PCBM film, the theoretical critical width differentiating local wrinkling and global buckling, according to Eq. (8), is ${w}_{cr}=393.99\,\mu m$. Here, $w=40\lambda =223.67\,\mu m$ and therefore $w < {w}_{cr}$. Based on ref. ^45^, local wrinkling is the stable form of instability under the given conditions. In another recent analytical study taking into account the effects of finite substrate thickness^55^, the critical condition separating global buckling and local wrinkling was established and shown in a graphical form. The graphical presentation suggests that the current numerical case is near the boundary between the local wrinkling and global buckling regimes. Our numerical modeling result points to the likely scenario that wrinkling and buckling can co-exist in this near-borderline case. Further discussion about this transitional feature is provided in a later section of this paper.

## Effects of displacement constraint at the bottom boundary

In all the numerical simulations presented thus far, the bottom boundary of the substrate remains unconstrained (Fig. 1c). An alternative treatment is to apply the “roller” boundary condition at the bottom, so displacement in the *y*-direction is prohibited but free tangential slide along the *x*-direction is allowed. One can expect that global buckling will be suppressed even for very thin substrates. The primary purpose of applying this boundary condition is to explore if and how the surface wrinkle features will be affected, compared to the traction-free condition.

**Figure 7:**
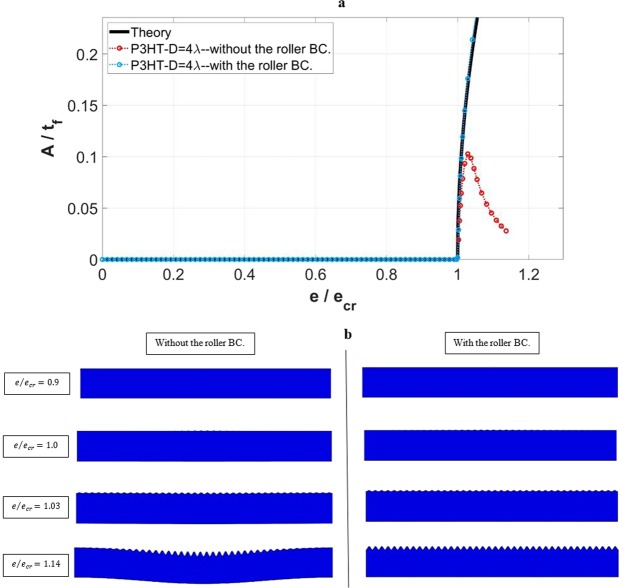
Numerically obtained mechanical response of the P3HT:PCBM film on the PDMS substrate, with *w* = 40*λ* and *D* = 4*λ*. (**a**) Normalized wrinkling amplitude (*A*/*t*_*f*_) as a function of normalized applied strain (*e*/*e*_*cr*_), including the theoretical response. (**b**) Deformed configurations for the two cases with and without the roller boundary condition at various applied strains near the onset of instability.

Consider again the case of P3HT:PCBM film with the problem geometry of *w* = 40*λ* and *D* = 4*λ*. As presented in Fig. 6b, this model shows the transitional instability with both local wrinkling and global buckling, when the bottom boundary is unconstrained. Figure 7a shows the normalized wrinkle amplitude as a function of normalized compressive strain, for the cases with and without the roller boundary condition. The theoretical response is also included. The deformed configurations for the two cases at various applied strains are shown in Fig. 7b. When the bottom boundary is not constrained, the surface wrinkles start to develop when the strain reaches the critical value. It can be seen in Fig. 7a that the initial evolution of amplitude agrees well with the theoretical prediction. However, global buckling soon commences which also affects the wrinkle pattern. Since the plotted amplitude in Fig. 7a is based on the right-most wrinkle adjacent to the boundary, its diminishing amplitude is visible (left column of Fig. 7b) and is also manifested in the anomalous amplitude drop in Fig. 7a. With the imposed roller boundary condition along the bottom, uniform surface wrinkles, once formed, remain in place (right column of Fig. 7b) and the increase in amplitude stays close to the theory (Fig. 7a).

**Figure 8:**
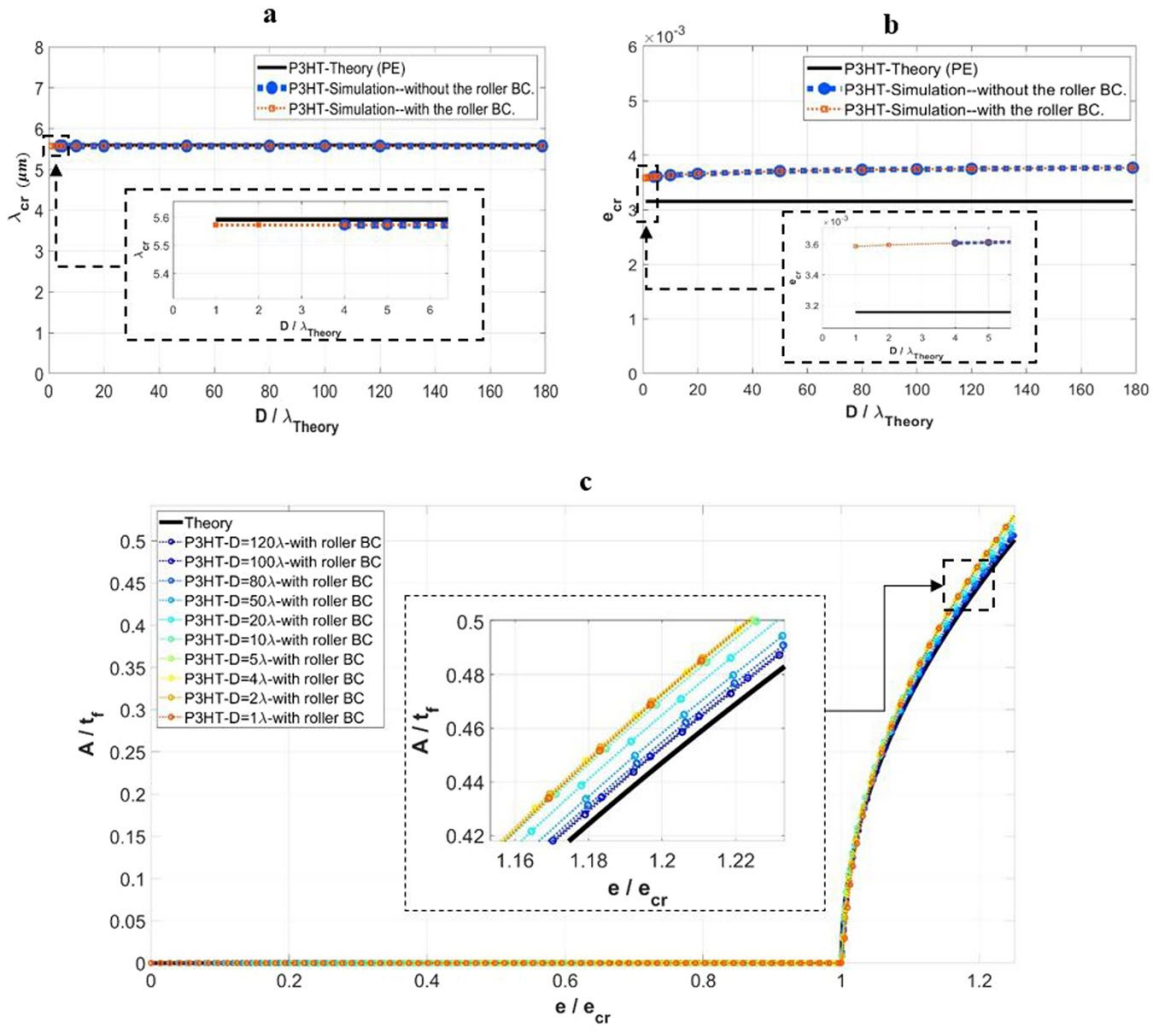
Effects of the applied boundary condition at the bottom of the substrate on the wrinkling parameters. (**a**) Simulated critical wrinkling wavelength (*λ*_*cr*_), and (**b**) critical wrinkling strain (*e*_*cr*_) as a function of normalized domain depth (*D*/*λ*). (**c**) Variation of the normalized wrinkling amplitude (*A*/*t*_*f*_) with normalized applied strain (*e*/*e*_*cr*_), with the roller boundary condition applied. The results pertain to the cases of *w* = 40*λ*, *D* ≥ *λ*, and the film material of P3HT:PCBM. Theoretical solutions are also included in the presentation.

A full set of numerical simulations was performed using the various domain depths and widths (as shown in Table 1). To conserve space only the results for the fixed width *w* = 40*λ* in the case of P3HT:PCBM film is presented here. Figure 8a,b show the simulated critical wrinkle wavelength and critical wrinkling strain, respectively, as a function of the domain depth (normalized by the theoretical wavelength). The theoretical values are also included as horizontal lines. As evidenced in the figures, constraining the bottom boundary by prohibiting the *y*-displacement results in the same critical wavelengths and critical strains as the unconstrained boundary. The numerical simulations yield a higher critical strain compared to the theoretical plane-strain solution, as mentioned previously. The evolution of the normalized wrinkling amplitude in response to the normalized applied strain, using the constrained bottom boundary for all the domain depths considered, are shown in Fig. 8c. Also included is the theoretical response based on Eq. (3). By comparing Fig. 8c with Fig. 4a, the amplitude is seen to also follow the same trend as the domain depth becomes smaller. In fact, the difference caused by the bottom boundary constraint on the wrinkling amplitude is negligible.

Overall, the results presented in Fig. 8 illustrates that, for the depth range of *D* ≥ 5*λ*, surface wrinkles form and the wrinkle configurations for both the constrained and unconstrained boundary conditions are essentially identical. For the depth range of *D* < 5*λ* (down to the smallest case considered, *D* = 1*λ*), the roller boundary condition suppresses global buckling and leads to the formation of surface wrinkles, and the wrinkling features are nearly identical to those of the thicker substrate without any bottom constraint. As a consequence, for the purpose of simulating surface wrinkling, one is able to use a much thinner model combined with the roller boundary condition to represent the thick geometry, thus significantly reducing the computation demand.

## Bilayer composite films

The wrinkling deformation instability of a bilayer film stack above a very thick substrate has been comprehensively analyzed in our previous study^37^. The problem domain was large, and many imperfections were distributed along the film-substrate interface for achieving a uniform wrinkle pattern. In this section, we present composite film results for a fixed domain width using only one imperfection and examine the instabilities resulting from various domain depths. Therefore, global buckling is now a possibility when the substrate thickness is sufficiently reduced.

There have been limited analytical/numerical studies on surface wrinkling in the multilayer film structures^56–60^. A short overview of the theories is presented first, focusing on the case of bilayer film where wrinkling occurs in tandem^61^. The theories are based on the analytical expressions for structures with a single-layer film, modified with the effective modulus of the bi-layer structure. The effective bending modulus *E*_*b*_ and the effective tension (uniaxial) modulus *E*_*t*_ can be expressed as^41,56,61^

9 $${E}_{b}=\left[\frac{1+{m}^{2}{n}^{4}+2mn(2{n}^{2}+3n+2)}{{(1+n)}^{3}(1+mn)}\right]{E}_{f2}$$

and

10 $${E}_{t}=\left(\frac{{t}_{f1}}{{t}_{f}}\right){E}_{f1}+\left(\frac{{t}_{f2}}{{t}_{f}}\right){E}_{f2},$$

where the subscripts *f*1 and *f2* represent the upper and lower film layers, respectively. The modulus ratio (*m*) and thickness ratio (*n*) are defined as *m* = (*E*_*f*1_/*E*_*f*2_), and $n=({t}_{f1}/{t}_{f2})$, and *t*_*f*_ is the sum of the film thicknesses (*t*_*f*_ = *t*_*f*1_ + *t*_*f*2_). Note that *E*_*t*_ is the longitudinal composite modulus following the simple rule-of-mixtures. By substituting Eqs. (9) and (10) into the single-film wrinkling parameters, Eqs. (1–3), the expressions of the wavelength ${\bar{\lambda }}_{cr}$, critical wrinkling strain ${\bar{e}}_{cr}$, and amplitude $\bar{A}$ for the case of bilayer films are then

11 $${\bar{\lambda }}_{cr}=2\pi {t}_{f}{\left[\frac{(1-{\nu }_{s}^{2}){E}_{b}}{3(1-{\nu }_{f}^{2}){E}_{s}}\right]}^{\frac{1}{3}},$$

12 $${\overline{({e}_{xx})}}_{cr}=\frac{1}{4}\left(\frac{{E}_{b}}{{E}_{t}}\right){\left[\frac{3(1-{\nu }_{f}^{2}){E}_{s}}{(1-{\nu }_{s}^{2}){E}_{b}}\right]}^{\frac{2}{3}},$$

13 $$\bar{A}={t}_{f}{\left[\left(\frac{e}{{e}_{cr}}-1\right)\left(\frac{{E}_{b}}{{E}_{t}}\right)\right]}^{\frac{1}{2}}.$$

The simulation results are now presented, based on the model depicted in Fig. 1c but with the thin film consisting of two materials, with the upper layer being P3HT:PCBM and the lower layer adjacent to the PDMS substrate being PEDOT:PSS. The bottom boundary of the substrate is unconstrained. The total film thickness is fixed at *t*_*f*_ = 0.1 *μm*, and an equal thickness percentage of 50% is considered for each film material (which is equivalent to the sub-layer thickness of *t*_*f*1_ = *t*_*f*2_ = 0.05 *μm*). Following the same procedure for the case of single-layer film, various models with the dimensions of $w=40\bar{\lambda }$ and $1\bar{\lambda }\le D\le 120\bar{\lambda }$ are considered for the numerical simulations. Only the salient results are presented below.

**Figure 9:**
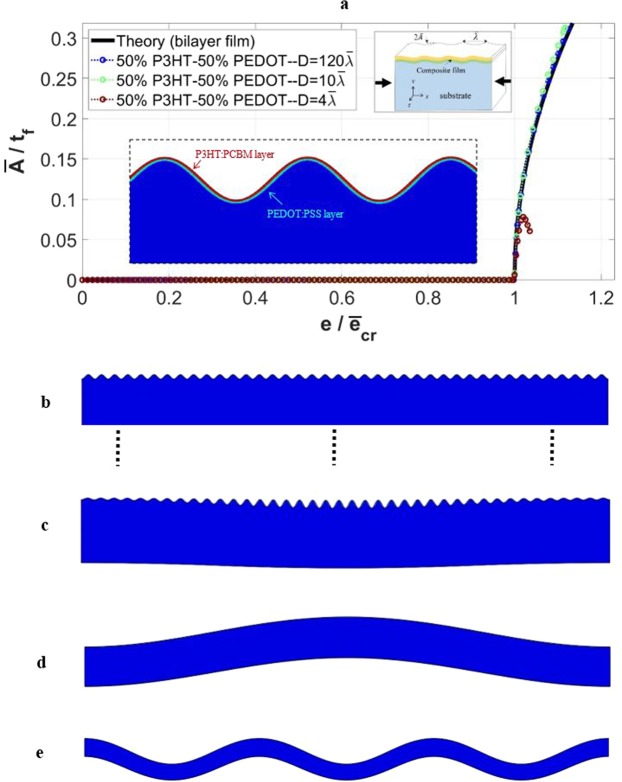
Simulation results for the case with bilayer films, with the width of $w=40\bar{\lambda }$ and various depths. (**a**) Variation of the normalized wrinkling amplitude ($\bar{A}/{t}_{f}$) with normalized applied strain $(e/{\bar{e}}_{cr}$), along with the theoretical response. (**b**–**e**) Numerically obtained instability modes for the domain depths of (**b**) $D\ge 5\bar{\lambda }$, (**c**) $D=4\bar{\lambda }$, (**d**) $D=2\bar{\lambda }$, (**e**) $D=\bar{\lambda }$.

Figure 9a shows the evolution of wrinkle amplitude as the applied strain progresses, for three different domain depths. The theoretical response included in the figure is based on Eq. (13). Figure 9b to e show the representative deformed configurations for the depths of $D\ge 5\bar{\lambda }$, $D=4\bar{\lambda }$, $D=2\bar{\lambda }$, and $D=\bar{\lambda }$, respectively. Figure 9a reveals that, similar to the single-layer film scenario, a thinner substrate results in slightly greater amplitudes. The anomaly displayed by the case $D=4\bar{\lambda }$ is again due to global buckling, in that surface wrinkles near the edges start to diminish once the central portion of the structure starts to buckle downward (Fig. 9c). This geometry also signifies the transitional instability where local wrinkling and global buckling co-develop. With a thicker substrate, Fig. 9b, only uniform surface wrinkles can be observed. For thinner substrates such as in Fig. 9d,e, surface wrinkles are no longer present and global buckling governs the deformation. The analyses of bilayer composite films presented in this section demonstrate the generality of the geometric conditions in dictating the dominant mode of instability.

## Further discussion

### Summary of instability modes

**Figure 10:**
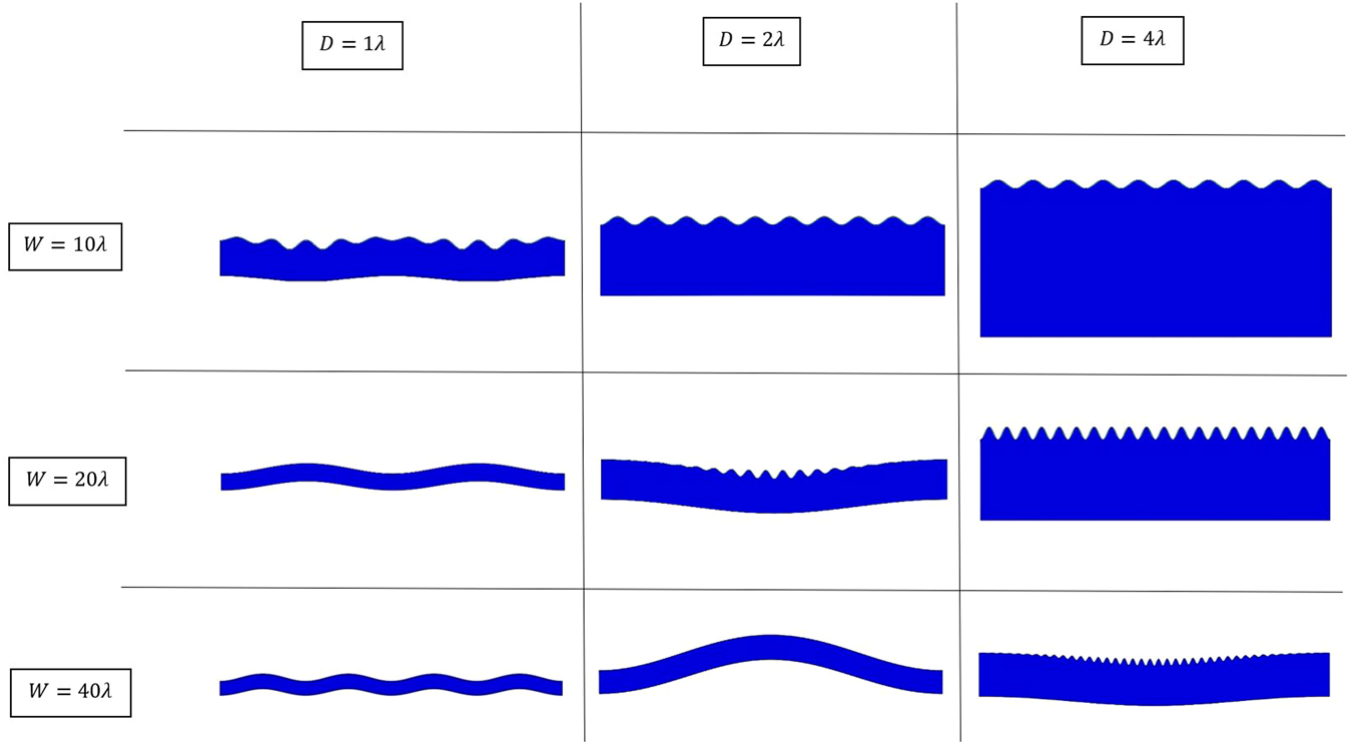
Various forms of numerically simulated instabilities resulting from the different domain widths and depths.

In the previous sections we presented extensive simulation results, all using one imperfection at the center of the film-substrate interface. This numerical approach offers a robust means to directly trigger instabilities. In response to the applied compression, the pre-instability deformation and post-instability deformation can both be directly obtained from a single modeling process. Figure 10 is a compilation of representative configurations showing the different forms of instabilities for the combinations of three domain widths and three domain depths. Although the total of nine cases in the figure is only a small subset of what was investigated (Table 1), they cover the range of width and depth which gives rise to all three forms of instabilities – surface wrinkling, global buckling, and transitional response between the two. The case of P3HT:PCBM film is used for the images in Fig. 10; similar appearances were also obtained for the PEDOT:PSS film.

It can be seen that a shorter (smaller *w*) and thicker (greater *D*) geometry results in the formation of pure surface wrinkles (upper-right region in Fig. 10). A longer (greater *w*) and thinner (smaller *D*) geometry favors pure global buckling (lower-left region in Fig. 10). The combination of *w* and *D* along the diagonal (upper-left to lower-right) shows the transition pattern where local wrinkles and global buckle co-exist. It is worth pointing out that these transitional cases all correspond to the relation of $\frac{w}{D}=\frac{N\lambda }{(N/10)\lambda }=10$ with *N* being an integer, which serves as a rough boundary separating the local wrinkling-dominant and global buckling-dominant regimes.

### Surface wrinkling in small models

**Figure 11:**
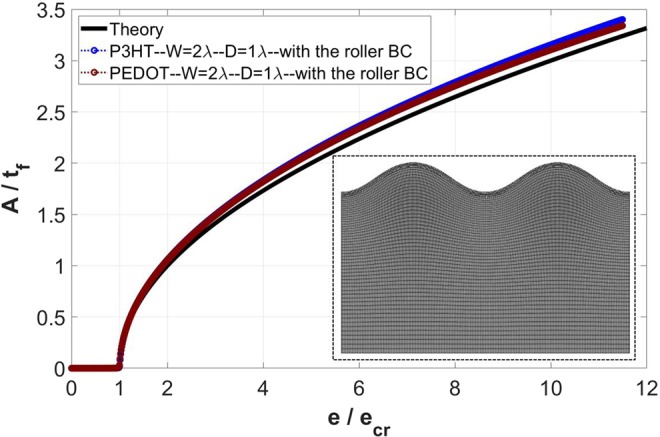
Variation of the normalized wrinkling amplitude (*A*/*t*_*f*_) with normalized applied strain (*e*/*e*_*cr*_), obtained for small models with *w* = 2*λ* and *D* = 1*λ*, for the two different single-layer films. Theoretical response is also included in the presentation. The substrate bottom is subject to the roller boundary condition. The deformed configuration for the case of P3HT:PCBM film at *e*/*e*_*cr*_ = 12.0 (including the finite element meshes) is shown in the inset.

The effects of domain width (*w*) and depth (*D*) were presented in the previous sections. In both sets of analyses, the current embedded imperfection approach was found to remain effective when *w* and *D* are reduced to very small values. However, in these sections the varying *w* (or *D*) was studied under large values of *D* (or *w*). Here we present the result when both *w* and *D* are small, namely *w* = 2*λ* and *D* = 1*λ*. Attention is devoted to surface wrinkles, and the bottom of the substrate is subject to the constrained roller boundary condition. Figure 11 shows the evolution of normalized wrinkle amplitude as a function of normalized applied strain. The figure includes both the cases of P3HT:PCBM single-layer film and PEDOT:PSS single-layer film. The finite element model in its entirety, after wrinkle formation, is shown as an inset. Note that this is an extremely small model, but it still gives rise to expected wrinkling features over a wide span of applied strain well beyond the critical point.

### Effects of displacement increment

The present study involves only isotropic linear elasticity. However, the static computational analysis takes into account geometric nonlinearity and the result is sensitive to the “time step” specified in the simulations. In the current context, “time step” is actually the displacement (or strain) increment. All the results presented thus far pertains to the sufficiently small increment which yields the “converged” deformation response. In this section we provide examples on how larger displacement increments may lead to different instability behavior.

**Figure 12:**
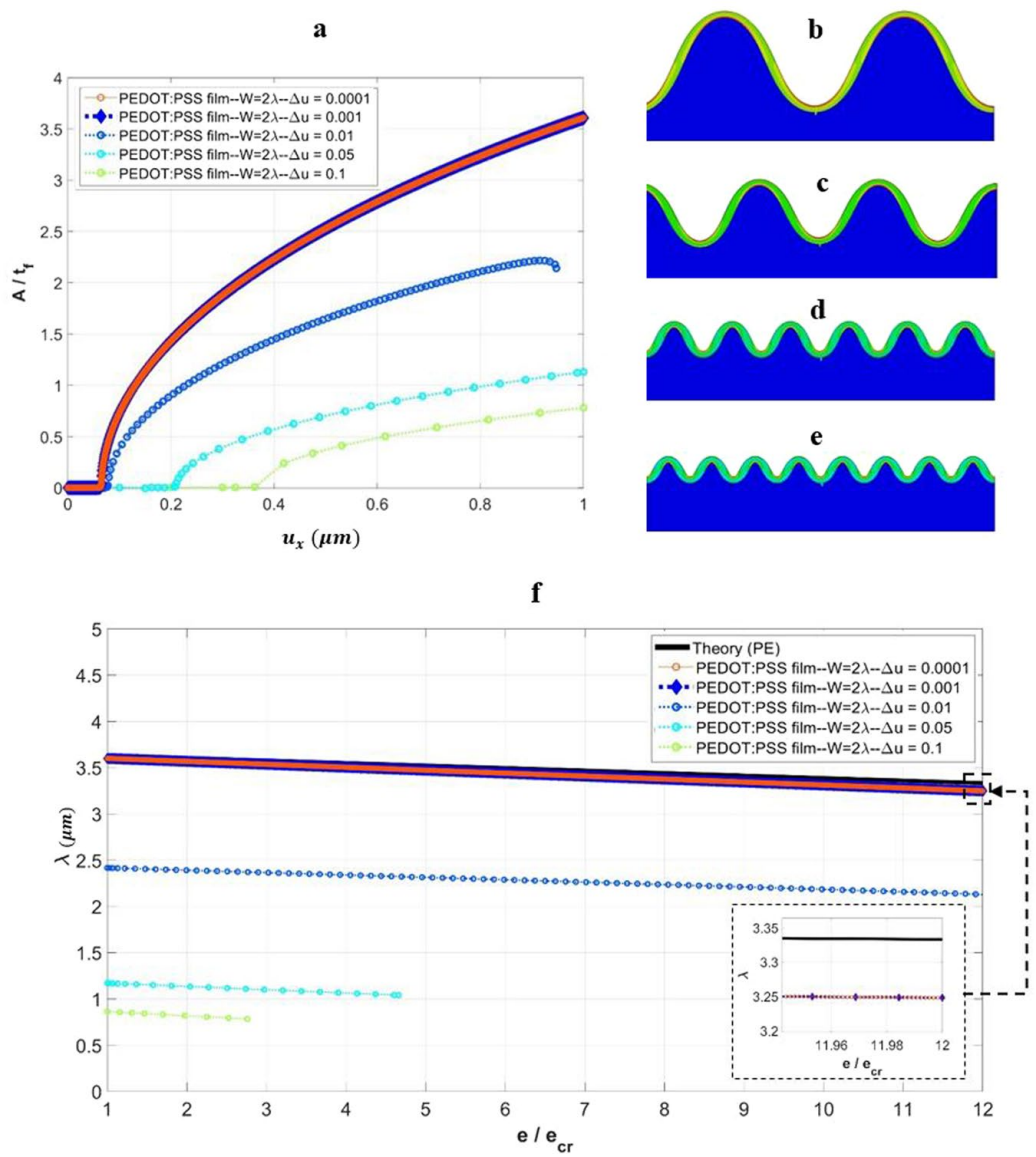
Effects of displacement increment on the surface wrinkling parameters for the model of PEDOT:PSS film with *D* = 2*λ* and *D* = 1000 *μm*. (**a**) Variation of the normalized wrinkling amplitude (*A*/*t*_*f*_) with the applied displacement (*u*_*x*_) under various displacement increments. (**b**–**e**) The numerically obtained wrinkle patterns for the various displacement increments of (**b**) $\Delta u=0.0001$ μm and 0.001 μm, (**c**) $\Delta u=0.01$ μm, (**d**) $\Delta u=0.05$ μm, and (**e**) $\Delta u=0.1$ μm (for better visualization a displacement scaling factor of 2 is used). (**f**) Variation of the wrinkling wavelength with normalized applied strain (*e*/*e*_*cr*_), under various displacement increments.

Consider first the PEDOT:PSS thin film above a thick PDMS substrate, with *w* = 2*λ* and *D* = 1000 *μm*. Several displacement increments are considered within the range of $0.0001\,\mu m\le \Delta u\le 0.1\,\mu m$, with otherwise identical simulation settings. (The equivalent strain increment range is $1.38\times {10}^{-5}\le \Delta e\le 0.0138$.) Fig. 12a presents the variation of normalized surface wrinkle amplitude with the applied displacement, obtained under the various displacement increments specified in the simulations. As can be seen, larger displacement increments cause different critical points and wrinkle sizes. For the two cases of smallest displacement increments considered ($\Delta u=0.0001\,\mu m$ and $\Delta u=0.001\,\mu m$), the numerical responses match fully. The wrinkle pattern associated with these two cases is shown in Fig. 12b, which also matches the analytical solution. The wrinkle patterns for the cases of $\Delta u=0.01,\,0.05,\,{\rm{and}}\,0.1\,\mu m$ are plotted in Fig. 12c–e, respectively. They all display smaller wavelengths and amplitudes. As a consequence, when applying the embedded imperfection approach, one should pay attention to the potential effects of the incremental size of the applied deformation.

Once wrinkles develop, their wavelength will become shorter as the applied compression increases. Figure 12f shows the evolution of wavelength as the applied compressive strain increases, for the various displacement increments considered. The theoretical response based on Eq. (4) is also included. A larger displacement increment is seen to result in a smaller wavelength right from the start. With sufficiently small increments ($\Delta u=0.0001\,\mu m$ and $\Delta u=0.001\,\mu m$), converged behavior is observed, and the initial (critical) wavelength matches the initial theoretical value. When the applied strain becomes very large (well beyond the critical point), the converged numerical solution falls slightly below the theoretical response, but the difference is small. Both the numerical and theoretical responses are linear; the slightly different slopes between the two cases may be attributed to the generalized plane-strain vs. plane-strain conditions.

The sensitivity of the numerical results to the specified displacement increment during the simulation is not limited to the surface wrinkling instability. In cases where the substrate is thin, global buckling was also found to be affected if the displacement increment is not sufficiently small. For instance, surface wrinkles may become dominant with larger displacement increments while the converged form of instability, under sufficiently small increments, is global buckle. Furthermore, the range of geometrical conditions under which concurrent surface wrinkling and global buckling develop was also found to be affected by the displacement increment used in the simulations. In what follows, we show an example where global buckling under small displacement increments become surface wrinkling when using a larger increment, and discuss their difference in terms of the evolution of internal stress field.

**Figure 13:**
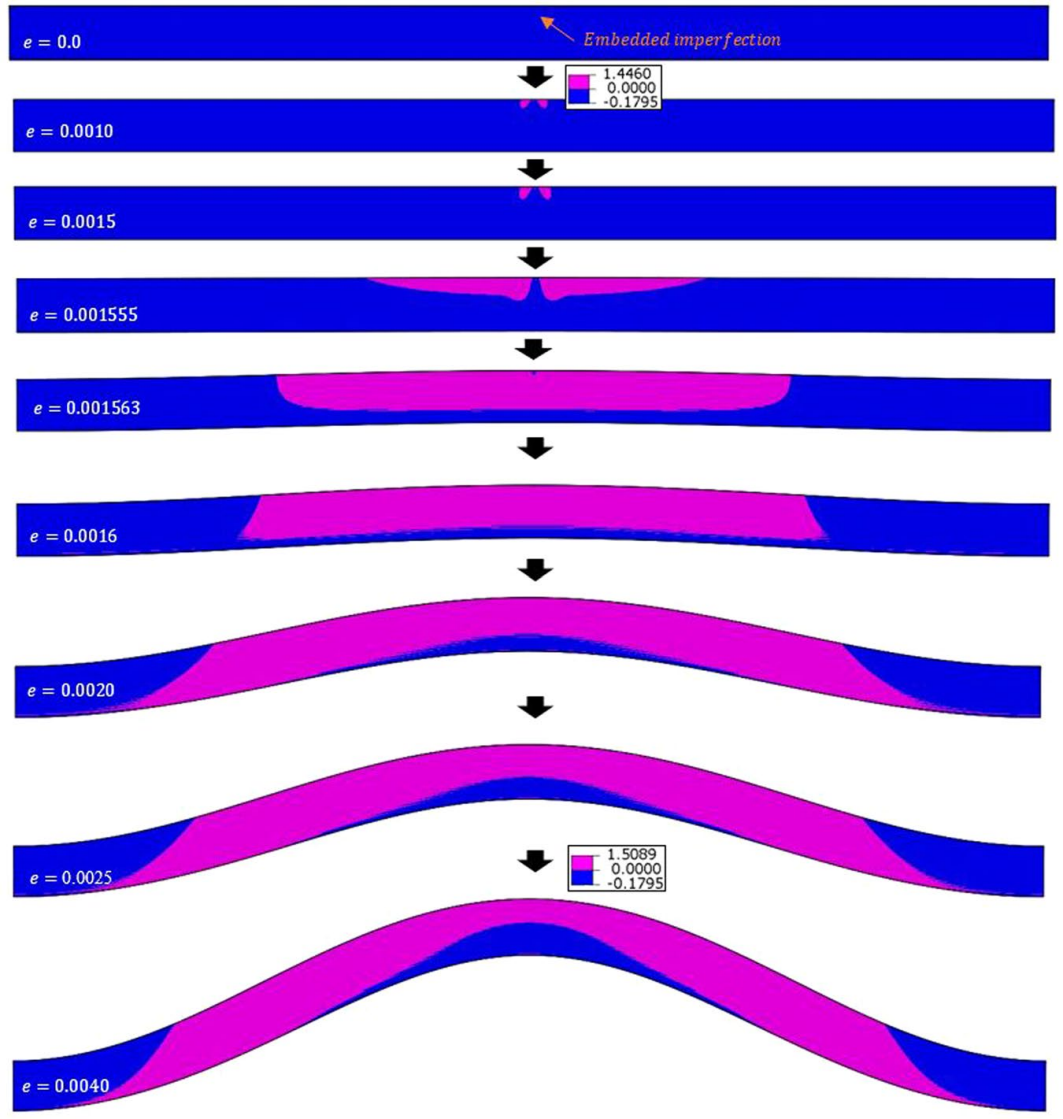
Progression of deformation at various compressive strains (*e*) for the problem of P3HT:PCBM film with *w* = 40*λ* and *D* = 2*λ*, under a sufficiently small displacement increment ($\Delta u=0.001\,\mu m$). The pink and blue colors represent, respectively, the tensile and compressive *σ*_*yy*_ stress fields (in MPa).

**Figure 14:**
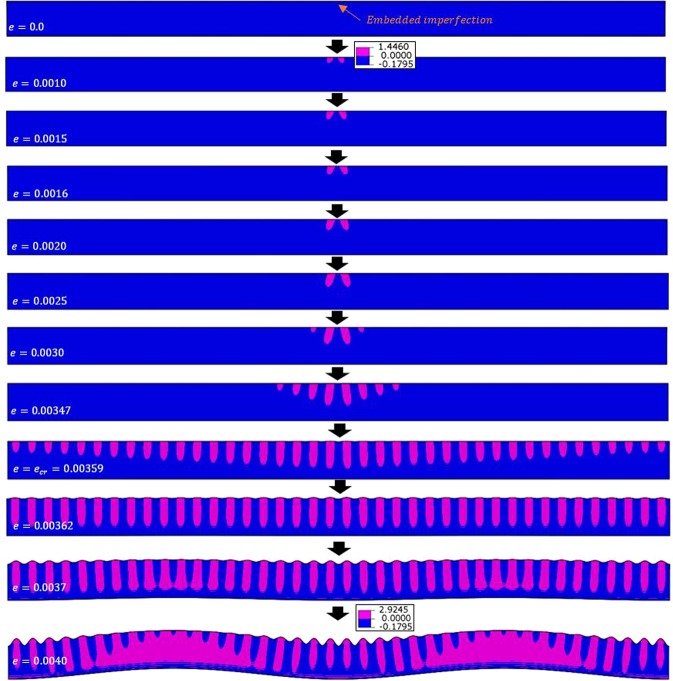
Progression of deformation at various compressive strains (*e*) for the problem of P3HT:PCBM film with *w* = 40*λ* and *D* = 2*λ*, under the displacement increment of $\Delta u=0.01\,\mu m$. The pink and blue colors represent, respectively, the tensile and compressive *σ*_*yy*_ stress fields (in MPa).

The case under consideration is P3HT:PCBM film with *w* = 40*λ* and *D* = 2*λ*. With a sufficiently small displacement increment ($\Delta u=0.001\,\mu m$) the converged form of instability is global buckling, as presented in Figs. 6c and 10 (bottom of the middle column). The progression of deformation at various compressive strains (*e*) is shown in Fig. 13, along with the contours of stress component *σ*_*yy*_. If the displacement increment of $\Delta u=0.01\,\mu m$ is used instead, a transitional behavior is then observed with surface wrinkles developed first followed by global buckling as shown in Fig. 14. The stress component *σ*_*yy*_ is chosen for Figs. 13 and 14 because both wrinkling and buckling involve deformation along the *y*-direction, and the *σ*_*yy*_ contour plot was found to be able to differentiate the two forms of instabilities better than the other stress and strain components. In the figures the pink color and blue color represent the tensile and compressive regions, respectively, with their boundary being the zero stress.

As can be seen, at the very beginning before the instability strain is reached, tensile regions emerge and grow in the vicinity of the imperfection. Up to the applied strain level of *e* = 0.0015, the stress contours are exactly the same in Figs. 13 and 14. Further compressive straining leads to completely different instability patterns and stress distributions. For a sufficiently small displacement increment as $\Delta u=0.001\,\mu m$ (Fig. 13), the tensile regions on the two sides of the imperfection continue to expand contiguously which is associated with the gradual formation of one large buckle. When $\Delta u=0.01\,\mu m$ (Fig. 14), the increment size appears to be too large to be accommodated by smoothly expanding the tensile region and thus multiple small tensile “bumps” are formed. These surface bumps continue to grow as the applied compression increases, and later become wrinkles. However, the tendency to form buckles still exists, which eventually leads to local wrinkles on top of global buckles as seen when *e* = 0.0037 and 0.0040. Note that in this case of coexisting wrinkles and buckles, the simulated critical wrinkling strain and wrinkle wavelength are still in agreement with the theoretical solutions.

It is worth emphasizing that the example given above does not imply that the transitional wrinkling-buckling behavior is just a pattern caused by larger displacement increments. All of the results presented in this paper before this section conform to the converged instability patterns under sufficiently small increments. As clearly illustrated in Figs. 6, 7, 9 and 10, local surface wrinkling, global buckling, and concurrent wrinkling-buckling are all possible outcomes depending on the combination of materials and geometry.

### Effects of imperfection size

**Figure 15:**
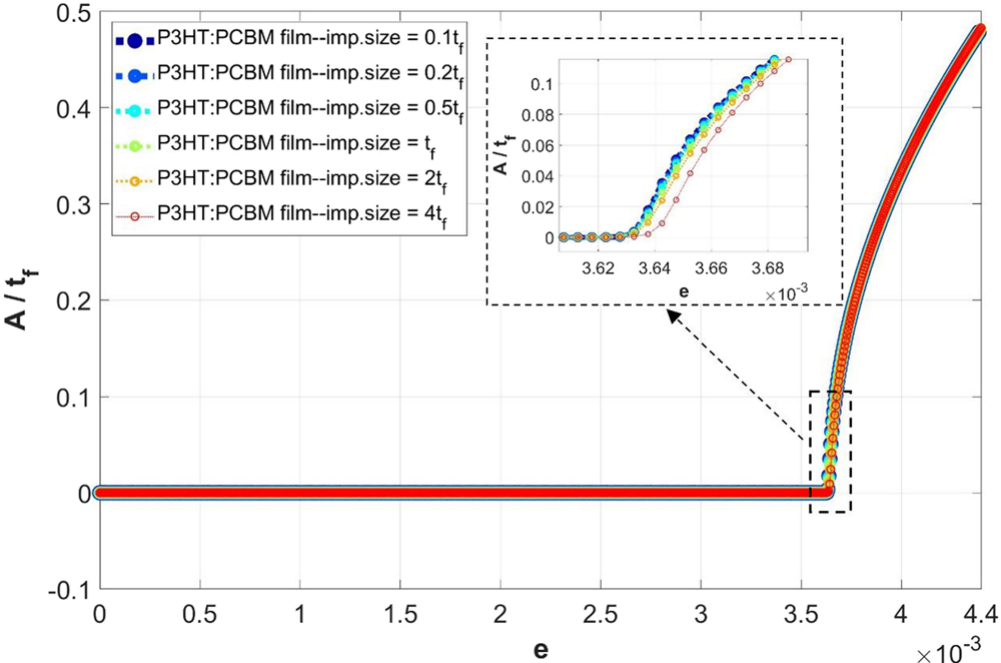
Variation of the normalized wrinkling amplitude (*A*/*t*_*f*_) with the applied compressive strain (*e*_*xx*_) with various imperfection sizes, for the P3HT:PCBM/PDMS model geometry of *w* = 40*λ* and *D* = 10*λ*, under a sufficiently small displacement increment ($\Delta u=0.001\,\mu m$). The inset shows the zoomed-in region around the bifurcation point.

It has been shown in our prior study that there is a high level of generality with the embedded imperfection approach, and the solutions are invariant over a wide range of imperfection properties and spatial distributions^37^. In the present work the periodic unit-cell approach based on placement of only one imperfection in the middle of film-substrate interface is proposed, which essentially eliminates the imperfection distribution dependency of the solutions. The effects of imperfection size are also studied and briefly discussed here. Note that the imperfection size refers to its vertical span (height) since its horizonal span is dictated by the converged finite element mesh size. Here we consider the P3HT:PCBM film with *w* = 40*λ* and *D* = 10*λ*, with a wide range of imperfection size from one-tenth to four times the film thickness. The numerical results are summarized in Fig. 15. In general, an imperfection size greater than two times the film thickness leads to localized (non-uniform) wrinkles near the imperfection location; however, theoretically verified uniform waves were seen at locations away from the imperfection. For the imperfection size greater than one-tenth and smaller than two times the film thickness, the simulated wrinkling results were independent of the imperfection size. If the imperfection size becomes as large as four times the film thickness, deviation of the simulated wrinkling amplitude starts to show as seen from Fig. 15. The numerical results are essentially imperfection-size insensitive within a wide range of imperfection size. It should be mentioned that the imperfection size was fixed at 0.5*t*_*f*_ in all the other simulations presented in this paper.

## Conclusions

The occurrence of instabilities in structures composed of a compliant substrate material with a thin film on top is studied numerically. Wrinkling of the thin film can be induced when the compressive strain attains a critical magnitude. Global buckling may also be triggered if the substrate is sufficiently thin compared to the specimen width. The current finite element-based computational approach utilizes an embedded imperfection in the model at the film-substrate interface. With no other special treatment of the model, the entire deformation history from pre-instability to post-instability can be numerically simulated in a seamless fashion. Over an extensive span of width and depth of the problem domain, the wrinkling features including wavelength, amplitude and critical strain were numerically characterized and compared with analytical solutions. Certain instability features such as critical strain cannot be accurately predicted by the theory due to the deviation from the strict plane strain behavior, but can be easily captured by the numerical model. For the purpose of simulating surface wrinkles in a thick structure, a very small model with a thin substrate is capable of generating accurate simulation results when a vertically constrained condition is applied to the bottom boundary of the substrate. In addition to the separate wrinkling and buckling instabilities developed under their respective geometric conditions, the current study establishes that concurrent surface wrinkling and global buckling can actually occur. While a converged instability pattern exists when a sufficiently small deformation increment size is used in the simulations, larger increments may result in different configurations. Correlating the local stress field with the overall shape change offers significant insight into the development of instability pattern. The comprehensive analyses presented in this paper, including the imperfection size effect, demonstrated the generality of the current embedded imperfection methodology and its advantages over other existing numerical approaches.

## Data Availability

All data generated and/or analyzed during the current study are included in this published article.
